# Circular RNAs in cancer: opportunities and challenges in the field

**DOI:** 10.1038/onc.2017.361

**Published:** 2017-10-09

**Authors:** L S Kristensen, T B Hansen, M T Venø, J Kjems

**Affiliations:** 1 Department of Molecular Biology and Genetics (MBG), Aarhus University, Aarhus, Denmark; 2 Interdisciplinary Nanoscience Center (iNANO), Aarhus University, Aarhus, Denmark

## Abstract

Circular RNA (circRNA) is a novel member of the noncoding cancer genome with distinct properties and diverse cellular functions, which is being explored at a steadily increasing pace. The list of endogenous circRNAs involved in cancer continues to grow; however, the functional relevance of the vast majority is yet to be discovered. In general, circRNAs are exceptionally stable molecules and some have been shown to function as efficient microRNA sponges with gene-regulatory potential. Many circRNAs are highly conserved and have tissue-specific expression patterns, which often do not correlate well with host gene expression. Here we review the current knowledge on circRNAs in relation to their implications in tumorigenesis as well as their potential as diagnostic and prognostic biomarkers and as possible therapeutic targets in future personalized medicine. Finally, we discuss future directions for circRNA cancer research and current caveats, which must be addressed to facilitate the translation of basic circRNA research into clinical use.

## Introduction

During the past decades, new cellular roles for RNA molecules have been continuously discovered leading to the current understanding that gene expression dynamics in cancer pathology are extremely complex. Recently, it has become apparent that thousands of genes produce highly conserved and stable covalently closed RNA circles with gene-regulatory potential. Although circular RNAs (circRNAs) were first discovered nearly 40 years ago,^1^ it was early on suggested that they may represent errors of the normal splicing process^2^ and were generally considered peculiarities of uncertain biological importance. In recent years, however, we have witnessed an explosion in published studies on all aspects of circRNA biology leading to the common understanding that these molecules are important players in normal cellular differentiation and tissue homeostasis as well as in disease development. Importantly, the expression of circRNAs does not often correlate well with the expression of host gene linear expression. This argues that circRNAs are not merely steady-state byproducts of mRNA splicing but rather the product of a new type of regulated alternative splicing. Another piece of evidence that circRNAs are biologically important has come from sequence conservation analyses.^3, 4^ Memczak *et al.*^4^ showed that 23 human circRNAs with orthologous exons being circularized in mice were more conserved in the third position of codons compared with exons not observed to be part of circRNAs, as expected if the circRNAs have important non-coding functions.

There are several reasons why these molecules lurked in the shadows for so many years. First, it is necessary to actively search for circRNAs to be able to detect them. Conventional reverse transcriptase-quantitative PCR (RT-qPCR) assays will not distinguish circular from linear RNA when using the linear genome as template for primer design. Likewise, sequencing reads spanning back-splicing junctions in deep sequencing data are discarded in standard bioinformatic pipelines as they do not map to the linear reference genome. Another feature of circRNAs that has facilitated their escape from the spotlight is the lack of poly(A) tails as most protocols for RNA sequencing library preparation include a poly(A) purification step to remove rRNA.

We were among the first to reveal a gene-regulatory function of a particular circRNA. We named it circRNA sponge for miR-7 (ciRS-7), as it contains >70 conserved binding sites for miR-7.^4, 5^ It is also known as CDR1 antisense (CDR1as), as it is located on the opposite strand of the gene *CDR1*. Owing to the relatively high expression and stability of ciRS-7 in many human tissues, it can increase the expression levels of miR-7 target genes by suppressing miR-7 activity. Because miR-7 directly targets several oncogenes and is involved in many different human cancers,^6^ ciRS-7 has now become one of the most studied circRNAs. Other circRNAs have subsequently been shown to have microRNA (miRNA) sponging properties, though most circRNAs are believed to possess other functions.^7^ Here we will discuss the various functions that have been proposed for circRNAs with an emphasis on their relevance in cancer.

**Figure 1:**
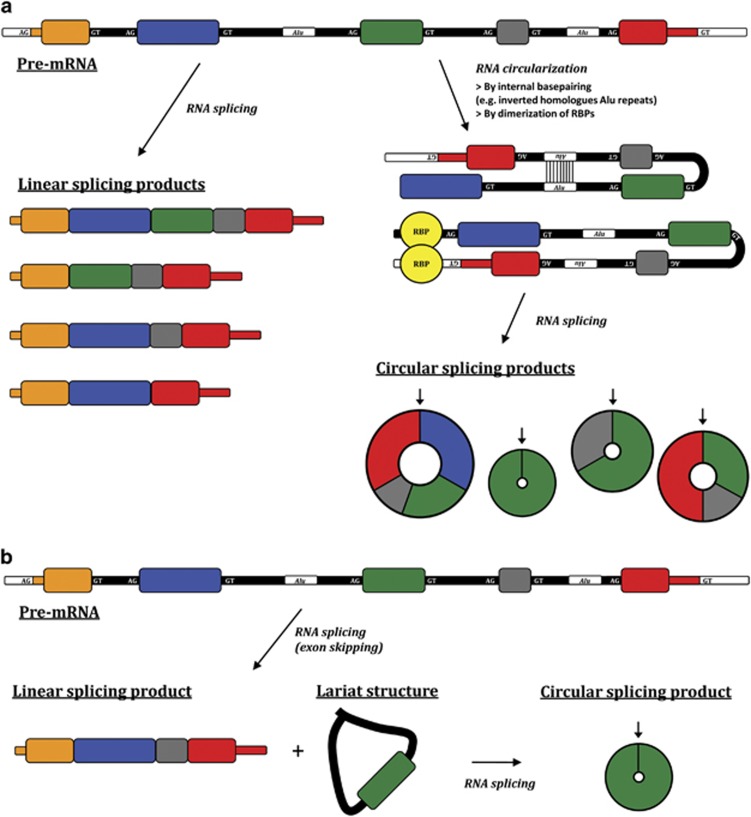
Mechanisms of circRNA biogenesis. (**a**) Efficient back splicing and circRNA generation requires that the 3′-end of an exon be located in close proximity to an upstream 5′-end of the same or another exon. This may be facilitated by the formation of a looping structure held in place by complementary base pairing of the RNA, for example, due to the presence of flanking reverse complementary *Alu* repeats. Alternatively, the looping structure may be held in place by binding of dimerizing RNA binding proteins with corresponding binding motifs present in the flanking regions. The small arrows indicate the back-splicing junctions of the circular splicing products. (**b**) Alternatively, circRNAs can be generated from an exon-containing lariat precursor created by an exon-skipping event followed by internal splicing depending on the kinetics of splicing versus debranching and exonuclease-mediated degradation.

Not much is known about how circRNA biogenesis is regulated. For some circRNAs, the characteristic back-splicing reaction that covalently joins the 3′-end of an exon to an upstream 5′-end, leading to the formation of a circRNA, is facilitated by reverse complementary *Alu* repeats flanking the circularized exon.^8, 9, 10, 11, 12^ Back splicing can also be regulated by splicing factors through their binding to *cis*-acting splicing-regulatory elements, although with regulatory rules distinct from those of canonical splicing.^13, 14^ Another mechanism that has been proposed for circRNA biogenesis involves an exon-containing lariat precursor created by an exon-skipping event. This lariat can be internally spliced leading to removal of the intronic sequence and production of a circRNA.^15^ Only a few transacting RNA-binding factors involved in circRNA biogenesis have been discovered. Most studied are ADAR, quaking (QKI), FUS, HNRNPL and DHX9, which regulate some but not all circRNAs.^10, 16, 17, 18, 19^ Interestingly, QKI has recently been shown to be a novel tumor suppressor in lung cancer and to be associated with prognosis,^20, 21^ and DHX9 has been suggested as a potential therapeutic target in several malignancies.^22^ A summary of circRNA biogenesis mechanisms is provided in Figure 1.

The regulation of circRNA turnover is also not well understood. High cellular stability is a common circRNA feature owing to the lack of free ends and resistance to exonucleolytic degradation, but circRNA may contain specific endonuclease sites that could be cleaved in a regulated manner. At least one example exists where a highly complementary site to miR-671 triggers specific cleavage of ciRS-7 by Ago2.^23^

Though only limited knowledge has been attained on the mechanisms governing the expression of these important biomolecules, hundreds of circRNAs have been shown to be deregulated in distinct human cancers, including liver, lung, colorectal, breast, prostate, bladder, ovarian, kidney and gastric cancer as well as hematological malignancies and tumors of the central nervous system. Thus it is becoming increasingly clear that circRNAs are important in cancer pathogenesis, and they are likely to affect several of the hallmarks of cancer.^24^

The expression of distinct circRNAs is often disease specific or predictive of outcome. Thus circRNAs have great potential as biomarkers in cancer.^25^ This potential is further substantiated by circRNAs being detectable in body fluids, such as blood and saliva, which is essential if they are to be used as non-invasive biomarkers.^26, 27, 28, 29^

Altogether, this review summarizes the current knowledge on circRNAs in relation to their biological implications in cancer development and progression as well as their potential as diagnostic and prognostic biomarkers and as therapeutic targets in future personalized medicine. Finally, we also discuss future directions for circRNA cancer research and current caveats, which need to be addressed to facilitate the translation of basic circRNA research into clinical use.

## Potential functions of circRNAs in cancer

**Figure 2:**
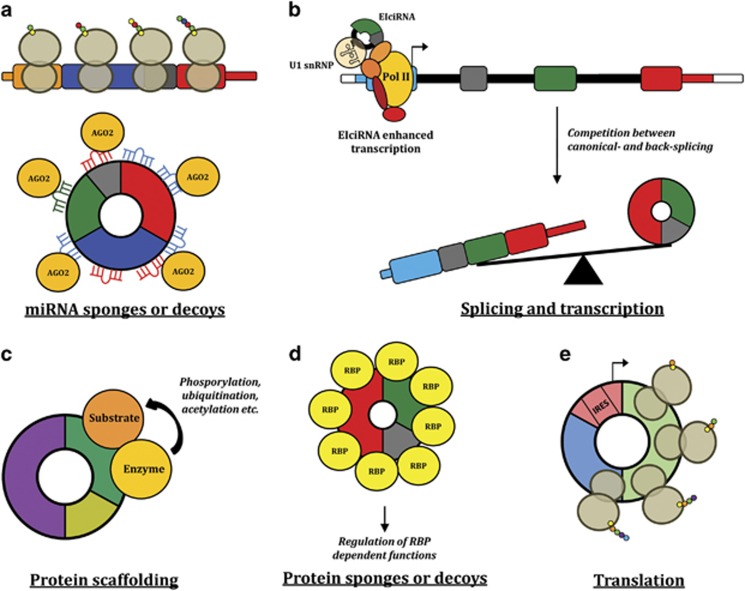
Putative functions of circRNAs in cancer. (**a**) Many circRNAs are likely to function as miRNA sponges or decoys. Binding of miRNAs to circRNAs may release target mRNAs from miRNA-dependent degradation resulting in more efficient translation. (**b**) Exon–intron circRNAs have been shown to associate with RNA pol II and enhance the transcription of their parental genes via interaction with U1 snRNP. Splicing and transcription of many genes may also be indirectly regulated through a competition between canonical splicing and back splicing. However, it is largely unknown which factors may impinge on the balance between circRNA and canonical linear splicing. (**c**) CircRNAs with binding motifs for an enzyme and its substrate may function as scaffolds facilitating co-localization and reaction kinetics. (**d**) CircRNAs with RNA-binding protein-binding motifs may function as sponges or decoys for proteins and thereby regulate their activity. (**e**) CircRNAs with IRES elements and AUG sites may, under certain circumstances, be used as templates for translation; however, it is currently unknown if this has any relevance in cancer.

Though some endogenous circRNAs contain AUG sites and internal ribosome entry site (IRES) elements, there is currently limited evidence for their translation *in vivo*.^3^ However, it has been shown that some circRNAs become translated in some tissues under certain conditions,^30, 31, 32^ but the relevance of this has yet to be shown in cancer. In general, circRNAs are expected to have other functions independent of their host genes, which may be related to the much longer half-life compared with linear RNA transcripts. Recently, a web tool named CircInteractome (circRNA interactome) has been developed for mapping RNA-binding protein- and miRNA-binding sites on human circRNAs, which may be a helpful first step toward understanding the function(s) of a circRNA in question.^33^ Below we discuss potential circRNA functions, which may explain their involvement in cancer (Figure 2).

### miRNA sponges or decoys

miRNAs are important players in the pathogenesis of virtually all human cancers.^34, 35^ Consequently, circRNAs that function as regulators of miRNA activity are likely to be involved in cancer. To date only a few circRNAs with multiple binding sites for a single miRNA have been discovered and it has been noted that most circRNAs probably have other functions than regulating miRNAs.^7^ The existence of circRNAs in organisms lacking RNA interference pathways also points toward other functions. However, it has been noted that miRNA-binding sites in circRNAs are virtually devoid of single-nucleotide polymorphisms, indicating that there is a strong selective pressure in conserving them,^36^ and multiple miRNA-binding sites may not be necessary in order to regulate miRNAs, as exemplified by transcribed pseudogenes^37^ and linear long noncoding RNAs^38^ functioning as miRNA decoys. Indeed, many circRNAs harbor single or a few miRNA-binding sites, and some harbor binding sites for multiple miRNAs or even regulate entire miRNA families. For instance, a circRNA derived from the *CCDC66* gene has been shown to harbor many more binding sites for miRNAs that target oncogenes than tumor-suppressor genes,^39^ and circFoxo3 harbor several miRNA-binding sites for miRNAs targeting the linear host transcript, thus releasing it from miRNA-dependent degradation.^40^ circHIPK3 is another example of a circRNA, which is likely to function as a miRNA sponge in cancer,^41^ and predicted miRNA-binding sites have been verified in Argonaute HITS-CLIP data for this circRNA.^42^ Finally, circPVT1 is an example of a circRNA sponging several tumor-suppressor miRNAs, including let-7b.^43, 44^

It should also be mentioned that binding of a miRNA to a circRNA might not always result in suppression of the miRNA. This is perhaps best exemplified by our work on ciRS-7 where we have observed it to be cleaved upon miR-671 binding in an AGO2-dependent manner and then releasing bound miR-7.^23^ Thus one could imagine that circRNAs with miRNA-binding sites may function as a reservoir of miRNAs or facilitate miRNA transportation. Nevertheless, more and more circRNAs are being designated miRNA-inhibitory functions in cancer (Table 1).

### Protein sponges or decoys

Some circRNAs with a high density of binding sites for a single or multiple RNA-binding proteins may function as protein sponges or decoys. The best experimentally supported example of a circRNA protein sponge is derived from the *mbl* locus, which harbors binding sites for the MBL protein itself.^9^ Thus MBL is prevented from binding to other targets when tethered to the circRNA. The MBL protein can bind to the introns flanking the circularized exon and facilitate its biogenesis and may therefore be part of an auto-regulatory circuit: when the protein is in excess, it may decrease the production of its own mRNA by promoting circRNA biogenesis. Another recent example is binding of HuR to a circRNA derived from the *PABPN1* gene.^45^ HuR also binds to *PABPN1* mRNA and enhances its translation. Thus extensive binding of circPABPN1 to HuR prevents HuR binding to *PABPN1* mRNA and lowers its translation. At this time, it is unknown whether circPABPN1 has a role in cancer, but it could be interesting to study as HuR is also targeting several mRNAs from tumor-suppressor and cancer-related genes, such as *TP53*, *VHL*, *MYC*, *HIF1A* and *BCL2*.^45^ Furthermore, HuR has been shown to regulate miRNAs, including the tumor-suppressor miRNA miR-7.^46^ In cancer, one could also imagine that circRNAs may bind tumor-suppressor proteins preventing them from carrying out their normal cellular tasks. However, strong evidence for this hypothesis is currently lacking.

### Protein scaffolding

circRNAs that harbor binding sites for enzymes and their substrates are likely to function as scaffolds facilitating contact between two or more proteins. This is perhaps best exemplified by circFoxo3, which has binding sites for mouse double-minute 2 (MDM2) and p53. Mutation of these binding sites or circRNA knockdown resulted in less pull-down of p53 with an MDM2 antibody and *vice versa* supporting the notion that circFoxo3 can function as a protein scaffold. Further, it was shown that circFoxo3 facilitated MDM2-mediated ubiquitination of p53, which subsequently becomes degraded by the proteasome.^47^ The long half-lives of circRNAs probably enhance their ability to function as scaffolds and more circRNAs functioning as such are likely to be discovered in the future.

### Splicing and transcription

Most circRNAs are located to the cytoplasm where they may function as miRNA or protein decoys or as transporters or scaffolds. However, some circRNAs are retained in the nucleus, where they may interfere with transcription or promote alternative splicing. Exon–intron circRNAs have been shown to associate with RNA pol II and promote the transcription of their parental genes via interaction with U1 snRNP.^48^ However, at this stage it is unknown whether exon–intron circRNAs become deregulated in cancer. On the other hand, there is a passive competition between circular and linear splicing for most host genes as circRNA generation is generally not compatible with formation of a functional mRNA. Thus perturbation of the balance between circular and linear splicing could promote aberrant transcription of oncogenes and tumor-suppressor genes. Finally, it could be imagined that some circRNAs become relocated to the nucleus as part of the malignant transformation as we have shown for circZEB1 in porcine brain development.^8^

## circRNAs and the hallmarks of cancer

**Figure 3:**
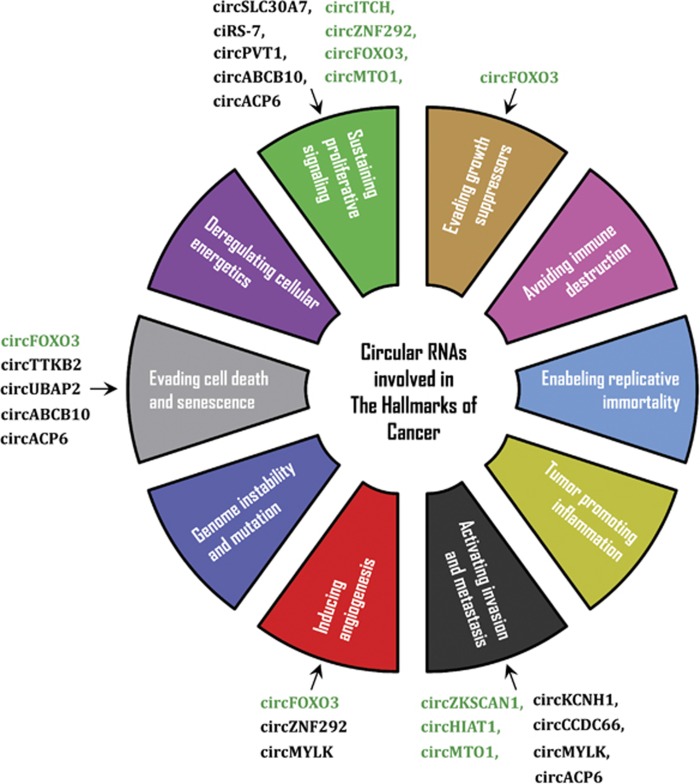
Circular RNAs involved in the hallmarks of cancer. Tumor-suppressor circRNAs are indicated in green and circRNAs with oncogenic properties are indicated in black.

At this time, circRNAs have been implicated in several of the hallmarks of cancer (Figure 3). One of the more studied circRNAs is derived from the tumor-suppressor gene *Foxo3* and has been found to promote cancer cell apoptosis through various mechanisms.^40, 47^ Du *et al.*^47^ showed that circFoxo3 may indirectly upregulate its linear host gene by interacting with p53 and MDM2. Overexpression of circFoxo3 decreased the interaction between Foxo3 and MDM2, releasing Foxo3 from MDM2-dependent degradation. This led to increased Foxo3 activity and, in turn, downstream Puma and Bax-mediated apoptosis.^47^ circFoxo3 has also been implicated in inhibition of angiogenesis^40^ and cell cycle progression,^49^ the latter by forming a circFoxo3–p21–CDK2 (cyclin-dependent kinase 2) ternary complex, which prevents CDK2 from promoting cell cycle progression.^49^ Unlike circFoxo3, circRNAs derived from *TTBK2* and *UBAP2* have been shown to inhibit apoptosis,^50, 51^ and similar to circFoxo3, circZNF292 has been shown to have tumor-suppressor properties by negatively regulating cell cycle progression.^52^ However, another study has suggested that circZNF292 is induced by hypoxia and exhibits proangiogenic activities.^42^ The molecular functions underlying these effects are unknown and it is unlikely to function as a miRNA sponge as Boeckel *et al.*^42^ found no evidence for Argonaute binding to circZNF292 from HITS-CLIP data. circMYLK is another example of a circRNA that may promote angiogenesis through the vascular endothelial growth factor A (VEGFA)/VEGFR2 signaling pathway.^53^

Another well-characterized circRNA is circITCH, which negatively affects the cell cycle and proliferation. These effects are mediated through inhibition of the Wnt/β-Catenin pathway.^54, 55^ On the other hand, ciRS-7 has a positive effect on the cell cycle by enhancing the epidermal growth factor receptor/RAF1/mitogen-activated protein kinase pathway through inhibition of miR-7 activity,^56^ and circSLC30A7 promotes cell cycle progression by enhancing CDK6 expression through sponging of miR-29b family members.^57^ circMTO1 was shown to negatively affect the cell cycle and proliferation by releasing p21 from miR-9 mediated downregulation.^58^ Finally, aberrant expression of several circRNAs have been associated with a late-stage diagnosis and metastases,^58, 59, 60, 61, 62, 63, 64, 65, 66, 67, 68^ and a few (circZKSCAN1, circCCDC66, circKCNH1 and circHIAT1) have been shown to affect metastasis both *in vitro* and *in vivo*.^39, 61, 65, 69^

It is likely that many more circRNAs are involved in the hallmarks of cancer as many studies have shown an effect of circRNA knockdown or overexpression on proliferation rates of cancer cells in culture without exploring the molecular mechanisms behind.

## Aberrant expression of circRNAs in diverse cancer types and tissues

Early on, it was shown that circRNAs are abundantly and differentially expressed in various cancer cell lines using publicly available high-throughput RNA sequencing (RNA-Seq) data from the ENCODE consortium.^70^ This, and other groundbreaking studies, has facilitated an explosion in published studies of circRNAs in cancer. Below we discuss the most important studies of circRNAs in solid tumors and hematological malignancies. The circRNAs most likely to be involved in cancer and their putative functions are shown in Table 1.

### Solid tumors

Studies of circRNA have been conducted in most common types of solid cancer. However, the majority were designed to investigate one or more predefined circRNAs without the possibility to discover novel circRNA species. Common for most studies is that circRNA expression is compared between cancer tissues and adjacent noncancerous tissues often followed by additional functional studies. Most of the studies were performed using microarray analysis followed by validation of selected circRNAs in larger cohorts by RT-qPCR.

#### In basal cell carcinoma (BCC)

A common cancer of the skin, Sand *et al.*^71^ discovered 23 upregulated and 48 downregulated circRNAs in BCC tissue samples relative to noncancerous epithelial skin samples. The two most downregulated circRNAs were hsa_circ_0022383 and hsa_circ_0022392, which are both derived from the *FADS2* gene. Likewise, the two most upregulated circRNAs were derived from the same host gene, *LINC00340*, encoding a long noncoding RNA likely to be involved in BCC.^72^

#### In bladder cancer

Zheng *et al.*^41^ found an overall upregulation of circRNAs, which is in contrast to what has been found in most other cancers. Similarly, a study by Zhong *et al.*^73^ found more upregulated than downregulated circRNAs in bladder cancer tissues relative to adjacent noncancerous tissue samples. Most interestingly, a circRNA derived from *TCF25* was observed to promote proliferation and migration of bladder cancer cells by sponging miR-103a-3p and miR-107.^73^ More recently, a circRNA from *MYLK* was shown to have oncogenic properties in bladder cancer by binding miR-29a and abolishing the endogenous suppressive effect on its target gene *VEGFA*, in turn, leading to epithelial–mesenchymal transition, angiogenesis and metastasis.^53^

#### In breast cancer

Nair *et al.*^74^ used RNA-seq data from 885 samples, including tumors and normal adjacent tissue, provided from The Cancer Genome Atlas (TCGA). Limitations of this study are that the RNA-seq libraries from TCGA were prepared using a poly(A) purification step to remove rRNA and most tumor samples did not have a paired normal adjacent tissue sample. However, 256, 288 and 411 tumor-specific circRNAs were found in triple negative (TN), estrogen receptor positive (ER+) and HER2-positive breast cancer subtypes, respectively.^74^ Another study found the putative tumor suppressor, circFOXO3, to be significantly downregulated in breast tumor samples relative to benign samples^47^ and this circRNA is likely to have a role in breast cancer progression.^75^ Finally, a circRNA from *ABCB10* was shown to be upregulated in breast cancer and knockdown of this circRNA *in vitro* suppressed proliferation of breast cancer cells and enhanced apoptosis.^76^

#### In colorectal carcinoma (CRC)

Bachmayr-Heyda *et al.*^77^ showed a global reduction of circRNAs in cancer cell lines and CRC tissues compared with adjacent noncancerous tissues. This reduction was negatively correlated to proliferation and the authors explain this by circRNAs accumulating in non-proliferating cells due to their high stability, while being distributed between daughter cells of proliferating cells.^77^ In contrast to most circRNAs, ciRS-7 was found to be upregulated in CRC, while a circRNA derived from the m^6^A methyltransferase gene *METTL3* was the most downregulated.^77^ Upregulation of ciRS-7 in CRC was later also shown by Weng *et al.*^56^ in a large study comprising a training cohort of 153 primary CRC tissues and a validation cohort of 165. High expression of ciRS-7 was associated with poor patient survival in both cohorts. In another study, using RNA-seq data, circRNAs were also found to be significantly downregulated in CRC.^41^ In contrast, Hsiao *et al.*^39^ found several upregulated circRNAs in CRC, among them a circRNA derived from the *CCDC66* gene, which may function as an oncogenic decoy by binding miRNAs that mainly target oncogenes. Zhang *et al.*^66^ also found more downregulated than upregulated circRNAs in CRC tissues relative to adjacent noncancerous tissues, and the most downregulated was derived from the *PTK2* tumor-suppressor gene. Finally, downregulation of a circRNA derived from *ITCH*^78^ and upregulation of circRNAs derived from *STIL*^60^ and *BANP*^79^ in 45, 30 and 35 CRC samples compared with adjacent noncancerous tissue samples, respectively, have been observed.

#### In cutaneous squamous cell carcinoma

Sand *et al.*^80^ discovered 143 upregulated and 179 downregulated circRNAs in cutaneous squamous cell carcinoma tissue samples relative to noncancerous skin samples. The two most downregulated circRNAs were the same two *FADS2* gene-originating circRNAs found to be most downregulated in BCC, in another study by Sand *et al.*,^71^ while the two most upregulated circRNAs were derived from the *LARP1B* gene.

#### In esophageal squamous cell carcinoma (ESCC)

circITCH has been studied^54^ in 684 ESCC cases and paired adjacent noncancerous tissue samples. Similar to the studies of this circRNA in CRC and lung cancer, it was observed to be downregulated. Another study indicated that circRNAs may be involved in the development of radiation resistance in ESCC. circRNA expression was compared between an ESCC cell line and a self-established radioresistant counterpart. Several circRNAs were observed to be upregulated and downregulated; however, circITCH was not among those.^81^ Finally, Xia *et al.*^63^ investigated the expression of a circRNA derived from exons 15 and 16 of the *PRKCI* gene in 51 cases of ESCC, finding it to be upregulated compared with adjacent noncancerous tissue samples and related to poor tumor differentiation and late Tumor, Node, Metastasis stage.

#### In gastric cancer

Both Zheng *et al.*^41^ and Chen *et al.*^44^ found more downregulated than upregulated circRNAs using RNA-seq. An interesting circRNA emerging from the latter study is circPVT1, which was upregulated in gastric cancer. Surprisingly though, high expression of circPVT1 was an independent predictor of better overall survival and disease-free survival. Thus it is unclear at the moment whether circPVT1 acts as a tumor suppressor or an oncogene. Another interesting finding is that circRNA expression could be a promising tool for predicting early recurrence after radical surgery of stage III gastric cancer.^82^ However, this study was limited by a short patient follow-up time. Finally, three independent studies from the same group found three different circRNAs from the *CNIH4*, *HIAT1* and *KIAA0907* genes to be downregulated gastric cancer tissues relative to adjacent noncancerous tissues.^59, 61, 62^

#### In glioma

Song *et al.*^83^ analyzed RNA-seq data from 46 gliomas and noncancerous brain samples and discovered thousands of circRNA candidates of which 476 were differentially expressed. Interestingly, a circRNA derived from the *VCAN* gene, which is implicated in gliomagenesis, was found to be upregulated in both oligodendroglioma and glioblastoma.^83^ Barbagallo *et al.*^84^ analyzed the expression of miR-671-5p in glioblastoma cell lines and primary patient samples and found it to be overexpressed and correlated with downregulation of ciRS-7. Finally, circZNF292 has been found to be downregulated in glioma^52^ and a circRNA derived from the *TTBK2* gene to be upregulated.^50^

#### In hepatocellular carcinoma (HCC)

Overexpression of ciRS-7 was shown to be a risk factor for hepatic microvascular invasion and was inversely correlated with miR-7 expression in a study including 108 patients.^85^ However, there was no significant difference in disease-free survival between patients with high and low ciRS-7 expression, respectively. Another study of a single candidate circRNA derived from the *SHPRH* gene found it to be significantly downregulated in 89 HCC samples relative to adjacent noncancerous tissues.^86^ Likewise, Yao *et al.*^65^ found a circRNA from the *ZKSCAN1* gene to be significantly downregulated in 102 HCC samples compared with adjacent noncancerous tissues. Shang *et al.*^87^ also discovered differentially expressed circRNAs in HCC samples relative to adjacent noncancerous tissues. They were subsequently able to confirm a significant differential expression of a circRNA derived from the *EIF4G3* gene in a validation cohort of 30 patients. Fu *et al.*^67, 88^ identified 527 differentially expressed circRNAs of which most were downregulated in HCC. Two of the most downregulated were associated with HCC clinicopathological characteristics and derived from *SMYD4* and *FAM53B*, respectively. Using RNA-seq, Zheng *et al.*^41^ discovered that a circRNA derived from exon 2 of *HIPK3* was differentially expressed in HCC. Recently, Han *et al.*^58^ identified a circRNA from the *MTO1* gene to be downregulated in HCC and associated with poor prognosis. Finally, Guo *et al.*^89^ found circITCH to be downregulated with a concomitant inferior overall survival in a large cohort of HCC patients.

#### In lung cancer

Wan *et al.*^55^ studied 78 patients and focused on a circRNA derived from the *ITCH* gene. This circRNA was downregulated in lung cancer tissues relative to adjacent noncancerous tissues and correlated with host gene expression. In another study by Zhu *et al.*,^68^ a circRNA from *ACP6* was found to be upregulated in adenocarcinoma and associated with Tumor, Node, Metastasis stage and lymphatic metastasis.

#### In oral squamous cell carcinomas

Chen *et al.*^57^ found many circRNAs to be differentially expressed between oral squamous cell carcinoma tissue and adjacent noncancerous tissues. Among these, an upregulated circRNA derived from the *SLC30A7* gene was shown to function as a sponge for several miR-29 family members and to act as a competing endogenous RNA regulating CDK6 expression.^57^

#### In osteosarcoma

Jin *et al.*^69^ studied a single circRNA candidate from the *KCNH1* oncogene. It was found to be upregulated in osteosarcoma patient samples and cell lines and to promote proliferation, invasion and metastasis *in vitro* and *in vivo*.^69^ In another study, Zhang *et al.*^51^ found a circRNA from *UBAP2* to be the most upregulated circRNA in osteosarcoma patient samples relative to adjacent noncancerous tissues. High expression of circUBAP2 correlated with inferior overall survival of the patients and it was shown to promote osteosarcoma growth and inhibit apoptosis both *in vitro* and *in vivo*.^51^

#### In ovarian cancer

Ahmed *et al.*^90^ analyzed RNA-seq data from primary and metastatic sites from three patients with stage IIIC ovarian cancer but did not include noncancerous tissues in the study. In total, 67 580 circRNA candidates were identified in the patient samples, and it was reported that more circRNAs were differentially expressed between primary ovarian tumor and metastases than mRNAs.^90^ Bachmayr-Heyda *et al.*^77^ observed a global reduction in circRNA expression in immortalized normal ovarian surface epithelium cell lines compared with ovarian cancer cell lines. This may be explained by the immortalized normal ovarian surface epithelium cell lines proliferating faster than the ovarian cancer cell lines, thus allowing less time for accumulation of long-lived circRNA species.^77^ Finally, Bachmayr-Heyda *et al.*^91^ found reduced levels of circRNAs in miliary compared with non-miliary tumors, but this could not be explained by differences in proliferation rates.

Finally, circRNA expression has also been studied in kidney clear cell carcinoma and prostate adenocarcinoma by RNA-seq.^41^ In prostate adenocarcinoma, circRNAs were found to be expressed at lower levels compared with adjacent noncancerous tissues, whereas the opposite was observed in kidney clear cell carcinoma.

### Hematological malignancies

Only a few studies of circRNAs using primary patient samples in hematopoietic malignancies have been published. In one of the inaugural studies of circRNAs in cancer, Salzman *et al.*^92^ not only aimed at discovering new chromosomal rearrangements in diagnostic bone marrow samples from childhood cases of hyperdiploid B-linage acute lymphoblastic leukemia (ALL) using RNA-seq but also investigated the possibility that the genome-specified exon order might be rearranged during RNA splicing. To their surprise, they found hundreds of genes that produced circRNAs in the ALL patient samples. These circRNAs were, however, also detected in Hela cells and in normal hematopoietic progenitors (CD34+), naive B-cells (CD19+) and in neutrophils. On the other hand, it has been observed that circRNAs derived from genes involved in B-cell differentiation and ALL (*JAK2*, *PAX5*, *IKZF1*, *ETV6* and *EBF1*) are predominantly present in ALL compared with normal leukocytes.^93^ Another recent study investigated circRNA expression in acute myeloid leukemia and found several deregulated circRNAs, including a circRNA derived from *WDR37*.^94^ Finally, Guarnerio *et al.*^95^ reported that circRNAs can be derived from transcription of fusion genes generated by chromosomal translocations. The authors found circRNAs derived from multiple tumor-associated translocations, including PML–RARA in promyelocytic leukemia and MLL-AF9 in acute myeloid leukemia, and named these fusion-circRNAs. This aberrant generation of circRNA is probably a result of distant, unrelated intronic sequences of the translocated genes containing complementary repetitive sequences, which favor new back-splicing events. Importantly, Guarnerio *et al.*^95^ studied the specific functions of fusion-circRNAs and found that they contribute to tumor progression by increasing cell proliferation and clonogenicity. Moreover, a circRNA derived from the MLL-AF9 fusion protected leukemia cells from the cytotoxic effects of cytarabine, a drug used for treatment of leukemia.

## circRNAs as therapeutic targets

As illustrated above, deregulation of circRNAs may influence proliferative signaling, epithelial-to-mesenchymal transition, angiogenesis, apoptosis or drug resistance and, in this manner, directly have a causative role in the development of cancer. So far, there has been no preclinical reports where circRNAs alone have been used as targets or as therapeutic vectors for cancer treatment but this scenario will likely change in the future. When designing circRNA therapeutics, there are some important issues to be considered. Targeting oncogenic circRNAs should preferably be carried out in a manner that does not interfere with linear mRNA expression. A feasible therapeutic route may be to target the unique back-splice junction of oncogenic circRNAs by exogenous delivery of siRNA fully complementary to this site. Alternatively, it may be possible to interfere with back splicing by introducing antisense oligonucleotides complementary to back-splice signals in the pre-mRNA, including flanking intronic *Alu* repeats or binding sites for trans-acting splicing factors involved in back splicing. To induce the expression of tumor-suppressor circRNA, exogenous expression can be accomplished by gene therapy where DNA cassettes designed for circRNA expression are delivered. circRNA expression is usually accomplished by cloning the corresponding exon(s) with accompanied splice sites into a region flanked either by an artificial long inverted repeat^5^ or by cognate flanking regions containing *Alu* repeats.^96^ In the future, we may also see applications for preformed circRNAs independent of nuclear splicing and export. However, care should be taken not to induce an interferon response by the introduction of foreign circRNA.^97^ For any of the above-mentioned strategies, a major challenge will be to target a majority of the malignant cells to avoid recurrence of the cancer.

## circRNA as therapeutic vectors

The unique cellular stability and capacity of circRNA to sponge miRNA and proteins may also place circRNA as a promising vehicle for delivery of therapeutics. Harnessing the circRNA with multiple binding sites for oncogenic miRNA and/or proteins may restore controlled proliferation of the cancer cells or induce apoptosis. As circRNAs are expressed from Pol II promoters, expression can be limited to certain cell types by using cell-specific promoters or even to malignant cells by disease-activated control elements. Furthermore, the sponging repertoire can include combinations of miRNA- and protein binding sites specifically designed to target specific oncogenic profiles. In this respect, it is an important observation that the genetic content of the circRNAs generally has little influence on their capacity to form circles as long as direct complementarity to miRNAs is circumvented (unpublished observations), as this is likely to cause cleavage of the circRNA via Ago2’s slicer function as observed for miR-671-mediated cleavage of ciRS-7. Finally, it has been reported that circRNAs, provided the right translation signals, can serve as templates for protein expression.^30, 31, 32^ Hence, including expression cassettes for tumor-suppressor proteins may turn circRNA into a Swiss army knife for cancer therapy.

## Future directions and conclusions

The study of circRNAs in cancer is still in its infancy, and a common standard for reporting and naming circRNAs is lacking. We recommend that future studies provide circBase IDs for the studied circRNAs and name them according to host gene name with the prefix ‘circ’. If the circRNA is not found in circBase, the genomic position should be provided. Unfortunately, circBase is currently static and it would be beneficial to the field if this database was regularly updated with new circRNA candidates as well as a continuous removal of false-positive circRNAs.

Currently, circRNA cancer research is also hampered by most publically available RNA-seq data sets related to cancer having been prepared using a poly(A) purification step to remove rRNA, including most samples in the TCGA. Moreover, most genome-wide studies of circRNAs have been performed using microarrays on a limited number of cancer and adjacent noncancerous tissue samples, and a thorough examination of the tumor cell content in the samples by an experienced pathologist is often lacking. Likewise, the large number of circRNAs that can be detected by RNA-seq or microarray analysis makes correcting for multiple testing a major problem in determining the significance of differentially expressed circRNAs. However, most studies have validated the differential expression of selected circRNAs, using RT-qPCR with divergent primers flanking the back-splicing junction, in larger cohorts. Likewise, many of the differentially expressed circRNAs have been shown to have oncogenic- or tumor-suppressor functions *in vitro* and *in vivo*. As most studies are retrospective, it would be interesting to see how well the best circRNA biomarker candidates perform in large prospective clinical trials.

For the discovery of novel circRNA species involved in cancer, more studies using an unbiased approach, such as RNA-seq, are called for. However, it is important to realize that the results are highly dependent on RNA quality (unpublished observations) and the strategy chosen for library preparation.^98^ In addition, a plethora of different bioinformatic algorithms for circRNA detection is now available.^99^ Some prediction algorithms require gene annotation lists and knowledge of exon–intron structures, while others work *de novo*. This requirement may reduce the number of false positives but may also preclude the detection of some circRNAs, including ciRS-7. We have recently found a dramatic difference between the output of different algorithms, emphasizing that the circRNA species detected are candidates that need further validation.^100^ Therefore, it may be advisable to run more than one circRNA prediction algorithm on RNA-seq data to minimize the number of false positives in the absence of a gold standard algorithm. Though RT-qPCR is a powerful technique for validating genome-wide data, there is a caveat that template switching may occur during reverse transcription,^92^ which may lead to false positive or biased results. In addition, exon repeats (that is, exon concatemers in linear mRNA) have been observed,^101^ which can only be distinguished from circRNA in an RT-PCR-based approach by RNase R treatment or polyA enrichment. Finally, appropriate normalization of RT-qPCR data is challenging^102^ and the use of a single, unvalidated reference gene may lead to unreliable conclusions. Thus Northern blotting is considered the gold standard for validation of novel circRNAs as no reverse transcription and amplification steps are part of the protocol.

*In situ* hybridization is another attractive approach toward quantification of circRNA expression, which is likely to be employed more in future circRNA cancer research. Especially because it provides spatial information about specific circRNAs and is compatible with formalin-fixed paraffin-embedded tissue samples. Thus it is possible to distinguish circRNA expression in cancer cells from background expression of non-malignant cells within the tumor. Future research should also explore the usefulness of novel technologies for circRNA detection such as nanopore RNA sequencing (Oxford Nanopore Technologies, Oxford, UK), which can potentially provide information on the entire circRNA and not just the back-splice junction, and the nCounter platform from NanoString Technologies (Seattle, WA, USA), which can quantify RNA molecules without amplification and reverse transcription.^103^

A causal link between many differentially expressed circRNAs and tumorigenesis is also currently lacking, and the field is somewhat biased by the hypothesis that circRNAs as a class function as miRNA sponges. A bioinformatics approach to identify putative miRNA-binding sites in circRNAs should be used only for generation of hypotheses and a starting point for further studies. Likewise, care should be taken to assess the stoichiometric relation between the miRNA-binding sites of a proposed sponge and the target sites of the miRNA, as a significant release of target repression may often require many competing target sites.^104^ This issue remains difficult to access when cohorts of cells with differential expression of both miRNA and circRNA are analyzed. However, future studies of single cells will help to resolve this question. Additionally, it should be noted that canonical 6-nt binding sites may compete for miRNA binding with a potency 20% of that observed for canonical 8-nt sites.^105^ Unfortunately, such stoichiometric relations and the nature of the miRNA-binding sites of proposed circRNA sponges have rarely been evaluated. Likewise, data from, for example, luciferase reporter assays and AGO-HITS CLIP experiments are relatively rare in circRNA cancer research. However, in light of recent studies, suggesting that the majority of circRNAs have other functions than being miRNA sponges,^7, 42^ other possible functions of circRNAs should be explored in future studies.

The field of circRNA cancer research has some similarities with the cancer epigenetic field.^106, 107, 108^ Here methylation and downregulation of many tumor-suppressor genes were discovered before much was known about what was causing these aberrant epigenetic changes. Only later, mutations in epigenetic regulators such as *DNMT3A*, *TET2*, *IDH1*, *IDH2*, *EZH2* and *ASXL1* were discovered and linked to aberrant epigenetic landscapes in cancer.^109^ At this time, we know nearly nothing about what causes the deregulation of circRNAs in cancer. Mutations in spliceosome genes, such as *SF3B1*, *SRSF2* and *U2AF1*, are prevalent in cancer where they influence alternative splicing^110^ and miRNA expression.^111^ Likewise, mutations in these genes may influence circRNA biogenesis but evidence is still lacking. Altered expression or mutation of other trans-acting factors, such as QKI,^16^ may also cause the differential expression of circRNAs observed in most human cancers. Recently, QKI was shown to be aberrantly expressed in lung cancer and associated with poor prognosis in two independent studies,^20, 21^ but none of these studies investigated circRNA expression. Thus it is tempting to speculate that the aberrant expression of circRNAs observed in cancer may, in part, be explained by genetic and/or epigenetic changes of genes involved in their biogenesis. Mutations in *cis* regulatory elements such as inverted repeats or protein-binding motifs could, in theory, also influence circRNA expression in cancer, but this is also still an open research question that could be pursued in future.

Another aspect of circRNA biology that has been largely unexplored is their susceptibility to become posttranscriptionally modified. Chemical modifications of RNA such as N6-methyladenosine, N1-methyladenosine, 5-methylcytosine, 5-hydroxymethylcytosine, pseudouridine (Ψ) and inosine-to-adenine editing are central in the control of coding as well as noncoding RNA activity and stability.^112^ Many of the writers and erasers of these modifications are mutated or aberrantly expressed in cancer. Thus it is likely that circRNA activity is hampered or enhanced in cancer if they become aberrantly modified.

If circRNAs function as important drivers of tumorigenesis or tumor suppressors, it is logical to speculate that loci producing these become amplified and deleted, respectively, in cancer. However, to our knowledge, only one study has investigated such relationships and found that the upregulation of circPVT1 in gastric cancer can be explained, in part, by genomic DNA amplification.^44^

Finally, placing circRNA expression in the context of other molecular alterations observed in cancer is also something future studies should aim for. A preliminary study found circRNA expression and *KRAS* mutations to be linked in lung and colorectal cancer,^113^ and another recent study found that the oncogenic transcription factor c-Myc to regulate the expression of a large number of circRNAs.^114^ However, the majority of the published studies have not investigated such potential correlations.

In conclusion, circRNA research is still in its infancy and their functional role in tumorigenesis is just starting to be elucidated. The glimpse uncovered so far makes circRNA a promising candidate not only as a valuable biomarker for cancer but also as potential targets or drugs in cancer therapy.

## Figures and Tables

**Table 1:** CircRNAs potentially involved in cancer and their putative functions

*circBase ID (Alias)*	*Putative function*	*Name of the host gene*	*Type of cancer*	*Upregulated/downregulated in cancer*	*References*
hsa_circ_0004277	miRNA sponge^a^	*WDR37*	AML	Upregulated	^94^
hsa_circ_0075828	miRNA sponge^a^	*LINC00340*	BCC	Upregulated	^71^
hsa_circ_0075825	miRNA sponge^a^	*LINC00340*	BCC	Upregulated	^71^
hsa_circ_0022383	miRNA sponge^a^	*FADS2*	BCC CSCC	Downregulated Downregulated	^71^ ^80^
hsa_circ_0022392	miRNA sponge^a^	*FADS2*	BCC CSCC	Downregulated Downregulated	^71^ ^80^
hsa_circ_0041103	miRNA sponge	*TCF25*	Bladder cancer	Upregulated	^73^
hsa_circ_0002768	miRNA sponge	*MYLK*	Bladder cancer	Upregulated	^53^
Not provided (circ-Foxo3)	Protein scaffolding	*FOXO3*	Breast cancer	Downregulated	^47^
hsa_circ_0008717	miRNA sponge	*ABCB10*	Breast cancer	Upregulated	^76^
hsa_circ_0001946 (ciRS-7)	miRNA sponge	*CDR1AS*	CRC CRC Glioma HCC	Upregulated Upregulated Downregulated Upregulated	^77^ ^56^ ^84^ ^85^
Not provided (hsa_circRNA_104700)	miRNA sponge^a^	*PTK2*	CRC	Downregulated	^66^
hsa_circ_0000069	Not investigated	*STIL*	CRC	Upregulated	^60^
Not provided (circCCDC66)	miRNA sponge	*CCDC66*	CRC	Upregulated	^39^
Not provided (*cir-ITCH*)	miRNA sponge	*ITCH*	CRC Lung cancer ESCC HCC	Downregulated Downregulated Downregulated Downregulated	^78^ ^55^ ^54^ ^89^
hsa_circ_0000523 (circ_006229)	Not investigated	*METTL3*	CRC	Downregulated	^77^
hsa_circ_0001346	Not investigated	*RNF13*	CRC	Downregulated	^77^
hsa_circ_0001793	Not investigated	*IKBKB*	CRC	Upregulated	^77^
hsa_circ_0070933	miRNA sponge^a^	*LARP1B*	CSCC	Upregulated	^80^
hsa_circ_0070934	miRNA sponge^a^	*LARP1B*	CSCC	Upregulated	^80^
hsa_circ_0067934	Not investigated	*PRKCI*	ESCC	Upregulated	^63^
Not provided (circPVT1)	miRNA sponge	*PVT1*	Gastric cancer	Upregulated	^44^
hsa_circ_0000190	Not investigated	*CNIH4*	Gastric cancer	Downregulated	^59^
hsa_circ_0000096	Not investigated	*HIAT1*	Gastric cancer	Downregulated	^61^
hsa_circ_0000140 (hsa_circ_002059)	Not investigated	*KIAA0907*	Gastric cancer	Downregulated	^62^
Not provided (circ_VCAN)	Not investigated	*VCAN*	Glioma	Upregulated	^83^
Not provided (cZNF292)	Not investigated	*ZNF292*	Glioma	Downregulated	^52^
hsa_circ_0000594	miRNA sponge	*TTBK2*	Glioma	Upregulated	^50^
hsa_circ_0001649	miRNA sponge^a^	*SHPRH*	HCC	Downregulated	^86^
hsa_circ_0001727	miRNA sponge^a^	*ZKSCAN1*	HCC	Downregulated	^65^
hsa_circ_0005075	miRNA sponge^a^	*EIF4G3*	HCC	Upregulated	^87^
hsa_circ_0000284 (circHIPK3)	miRNA sponge	*HIPK3*	HCC	Upregulated	^41^
hsa_circ_0007874	miRNA sponge	*MTO1*	HCC	Downregulated	^58^
hsa_circ_0004018	miRNA sponge^a^	*SMYD4*	HCC	Downregulated	^67^
hsa_circ_0003570	Not investigated	*FAM53B*	HCC	Downregulated	^88^
hsa_circ_0013958	miRNA sponge	*ACP6*	Lung Cancer	Upregulated	^68^
hsa_circ_0016347	miRNA sponge	*KCNH1*	Osteosarcoma	Upregulated	^69^
Not provided (circUBAP2)	miRNA sponge	*UBAP2*	Osteosarcoma	Upregulated	^51^
hsa_circ_0013339	miRNA sponge	*SLC30A7*	OSSC	Upregulated	^57^

Abbreviations: AML, acute myeloid leukemia; BCC, basal cell carcinoma; circRNA, circular RNA; CRC, colorectal carcinoma; CSCC, cutaneous squamous cell carcinoma; ESCC, esophageal squamous cell carcinoma; HCC, hepatocellular carcinoma; miRNA, microRNA; OSCC, oral squamous cell carcinoma.

a Not validated experimentally.
